# A Latent Profile Analysis of Violent Offenders Based on PCL-R Factor Scores: Criminogenic Needs and Recidivism Risk

**DOI:** 10.3389/fpsyt.2019.00627

**Published:** 2019-09-06

**Authors:** Robert Johann Bernhard Lehmann, Craig S. Neumann, Robert Douglas Hare, Jürgen Biedermann, Klaus-Peter Dahle, Andreas Mokros

**Affiliations:** ^1^Medical School Berlin, Berlin, Germany; ^2^University of North Texas, Denton, TX, United States; ^3^University of British Columbia, Vancouver, BC, Canada; ^4^Hochschule der Polizei des Landes Brandenburg, Oranienburg, Germany; ^5^University of Hildesheim, Hildesheim, Germany; ^6^FernUniversität in Hagen, Hagen, Germany

**Keywords:** PCL-r, recidivism, criminogenic needs, risk assessment, psychopathy, LSI-R, subtypes

## Abstract

Clinicians and theorists have often proposed the two psychopathic subtypes of “primary” and “secondary” psychopathy, with recent research indicating some empirical support for both psychopathy subtypes, though the findings across studies are far from uniform. For the current study, latent profile analysis was used to investigate if homogeneous latent classes exist within a sample of 215 adult male violent offenders from Berlin, Germany. The age of the offenders at the time of the index offense ranged from 19 to 59 years. The results indicated a solution with four latent classes, which we refer to as *prototypical psychopaths* (LC1), *callous-conning offenders* (LC2), *sociopathic or dyssocial offenders* (LC3), and *general offenders* (LC4). Validation of the four subtypes involved examination of differences on recidivism risk; criminogenic needs; and general, violent, and sexual reoffending. The results also are discussed in terms of the issue of treatment amenability.

Clinicians and theorists long have proposed numerous psychopathic subtypes [see reviews in Refs. (1, 2)]; (Mokros, Hare, Neumann, & Habermeyer, in press). An early distinction offered by Karpman (3, p. 46) was between two forms of primary or idiopathic psychopaths who shared similar motivations and dynamics but differed in their interactions with others: *aggressive/predatory* and *passive/parasitic*. Similarly, Arieti (4, pp. 307–308) described several kinds of “true” psychopaths who differed from one another in their interpersonal and aggressive behaviors: the *simple* and the *complex* psychopath. Karpman’s aggressive/predatory and passive/parasitic variants can be viewed as analogous with Arieti’s simple and complex variants, respectively (5–7). Karpman, Arieti, and other early influential clinicians also described individuals with some features of psychopathy (primarily disinhibition, externalizing) but falling outside of the psychopathy construct. The terms for these individuals included secondary, symptomatic, or pseudo-psychopathy. As put by Mokros and colleagues (6, p. 273), “A common view was that psychopathy is rooted in genetic predispositions and social/environmental forces that are quite different from those that lead to secondary psychopathy. In this sense, diagnostic labels, such as secondary or symptomatic psychopathy, are problematic and misleading because they imply that individuals so labeled are psychopaths in the traditional sense of the term (8).” More appropriate terms for these individuals might be *sociopaths*, as described by Lykken (9), or dyssocial individuals who are not socialized in the usual sense and are antisocial with respect to society but loyal to members of their own group (6, p. 373). Paralleling the clinical descriptions, more contemporary theorists (10, 11) differentiated between primary psychopaths with a congenital affective “deficit” (i.e., genotype) and secondary psychopaths who did not develop basic affective competence due to traumatic interpersonal experiences (i.e., phenotype).

There is a considerable body of empirical literature on the topic of subtypes of psychopathy (2, 12), but it reflects studies that used a variety of different samples (e.g., correctional, treatment, or community samples), selection criteria (e.g., unselected samples versus extreme manifestations of psychopathy), and analytical techniques [e.g., cluster analysis, latent class analysis (LCA)]. Furthermore, psychopathy was defined and measured in different ways in these studies: Some researchers relied on self-report questionnaires, whereas others used clinical observer ratings primarily based on the Psychopathy Checklist—Revised (PCL-R; 13) or its derivatives. As measured with the PCL-R, psychopathy is a dimensional construct underpinned by four correlated first-order factors commonly referred to as facets (Interpersonal, Affective, Lifestyle, and Antisocial) constituting the two originals factors: Factor 1 (comprising the facets Interpersonal and Affective) and Factor 2 (comprising the facets Lifestyle and Antisocial). Given the diversity of approaches to the topic, subtyping studies have identified between two and four interpretable psychopathic subtypes (6, 14–16). For example, cluster analysis of the PCL-R facet scores of male offenders with high psychopathic trait levels resulted in four subgroups, or variants: prototypical (or primary) psychopaths, macho psychopaths, manipulative psychopaths, and pseudo- (or secondary) psychopaths (17). Mokros and colleagues (6) used latent profile analysis (LPA) with a large sample of male offenders (*N* = 1,451) with high PCL-R scores (≥ 27) and identified three latent classes labeled *manipulative psychopathy* (LC1), *aggressive psychopathy* (LC2), and a *sociopathic* or dyssocial subgroup (LC3). They (6, p. 372) suggested that “LC1 and LC2 represent phenotypic variations on the theme of psychopathy,” corresponding, respectively, to Karpman’s passive/parasitic and aggressive/predatory psychopathy, Arieti’s complex and simple psychopathy, Book and Quinsey’s (18) *cheater* and *warriorhawk* psychopathy, and the emotionally stable and aggressive psychopaths described by Hicks and colleagues (14). LC3 formed a separate subgroup consistent with conceptions of antisocial personality disorder and sociopathy. These findings were replicated with an independent sample of 487 male offenders (6). In a supplemental analysis, Mokros and colleagues (6) (http://dx.doi.org/10.1037/abn0000042.supp) raised the PCL-R threshold for inclusion in the LPA to 30+ (*n* = 856). Two latent classes emerged, virtually identical with LC1 and LC2, described above.

Despite some differences in findings, the subtyping studies generally have identified a subtype reflecting the traditional clinical construct of psychopathy (6, 15, 19, 20). Also, the majority of the studies have identified a group of “secondary psychopaths,” who tend to show higher scores on measures of anxiety (15, 20), self-reported antisociality, and childhood trauma (20) than do primary psychopaths.

Several authors (14, 21) consider elevations on Factor 1 of the PCL-R as indicative of primary psychopathy and high scores on Factor 2 as indicative of secondary psychopathy. However, the view that primary and secondary psychopathy map onto PCL-R Factors 1 and 2, respectively, is simplistic and inconsistent with clinical accounts of psychopathy (4, 22) and with empirical evidence that many of the features measured by Factor 2 (e.g., externalizing behaviors) are essential components of the psychopathy construct (23, 24). In particular, the Mokros et al. (6) study of offenders with extreme elevations of the PCL-R provided clear evidence of a primary subtype that displayed very high scores on the antisocial facet. At the same time, this study only focused on offenders at the very top of the distribution with extreme PCL-R scores and did not examine a sample of offenders who manifested the full range of PCL-R scores. Thus, an open area of research concerns the nature of the subtypes that may emerge when the entire range of PCL-R scores are employed for subtyping in a large sample of offenders [though see Ref. (5), for initial work in this area] (7). Here, Neumann et al. (7) as well as Krstic et al. (25) used total, unselected offender samples and identified four subtypes: prototypic (high scores on all four facets), callous-conning (elevated Interpersonal and Affective facet scores), sociopathic (elevated Lifestyle and Antisocial facet scores), and general offender (relatively low scores on all four facets). Validation analyses by Krstic et al. (25) using offense behavior showed prototypic subtype offenders to be more violent in the commission of their sexual crimes (compared to all other three subtypes) and general offenders to engage in more sexual behavior (compared to sociopathic offenders). These results indicate that the use of a total sample provides evidence of a range of subtypes, which may also vary considerably in their recidivism risk, criminogenic needs, and response to treatment. This is very important, as the challenging intermediate-level cases (e.g., callous-conning, sociopathic) will be encountered more frequently in general offender populations than extremely psychopathic offenders (i.e., prototypic).

## Risk, Need, and Recidivism

Given the clinical, theoretical, and empirical conceptualization of psychopathic and non-psychopathic subtypes, it is reasonable to think that they may differ in their risk, needs, or response to treatment. In fact, differences among empirically identified subtypes may help to highlight important risk and protective factors associated with individuals who display certain profiles of psychopathic features. To assess offender risk and needs, evaluators commonly use purpose-built dynamic risk assessment instruments, such as the *Level of Service Inventory—Revised* (LSI-R) (26), which assess constructs (i.e., criminogenic needs) that are both theoretically and empirically relevant for criminal conduct (27) and may be amenable to intervention. Generally, Simourd and Hoge (28) found that psychopaths (PCL-R score ≥30) scored significantly higher than non-psychopaths on several risk and needs areas as assessed by the LSI-R. Thus, the criminogenic need profiles of different psychopathic and non-psychopathic subtypes could help, for example, to identify the type of treatment that would be required to reduce recidivism risk. Since criminogenic needs include developmental factors (e.g., poor parenting, delinquent subculture), certain subtypes are likely to score higher on such factors, which may make them more amenable to treatment (29). In this regard, Poythress et al. (20), as well as Olver and colleagues (19), found some indication that secondary subtypes are more amenable to treatment (i.e., fewer unexcused absences, higher treatment motivation) than are primary subtypes. Poythress and colleagues (20) as well as Olver and colleagues (19) found no significant differences between primary and secondary psychopaths in terms of general or violent recidivism, but Olver and colleagues (19) did find significantly higher recidivism rates for sexual offending for secondary psychopaths.

## Purpose of This Study

This person-oriented study is divided into two parts. In the first part, we used LPA with a complete sample of violent offenders to determine if a manifest psychopathic subtype could be differentiated from other offender subtypes. Based on the theoretical literature and previous empirical findings (7, 25), we expected to find four viable latent classes (or profiles) of PCL-R facet scores. In accordance with the findings from North American and Swedish samples of adult male offenders (5, 7), we expected to find a class indicative of primary psychopathy with high average scores on all four PCL-R factors, a sociopathic class with low average scores on Factor 1 and high average scores on Factor 2, a class indicative of callous-conning offenders (with high average scores on Factor 1 and low average scores on Factor 2), and a general offender class with average low scores on all factors.

In the second part of the study, we sought to extend the literature on person-centered approaches (compared to variable-centered findings) in psychopathy research. Therefore, we sought to determine whether the latent classes thus identified differed in meaningful ways from one another with respect to the average recidivism risk for different offense types. In terms of recidivism, we expected sociopaths (“secondary” psychopaths) to be at risk for both general and violent recidivism. However, the subtype of primary psychopaths should display the highest overall recidivism rate. Moreover, we wanted to investigate the relationship between sexual recidivism and psychopathy subtypes in more detail. Finally, we examined which specific criminogenic need factors differentiated the psychopathy subtypes. Criminogenic needs were assessed by the LSI-R (26). Due to the expected elevation on Factor 2 of the PCL-R, we predicted the primary and sociopathic subtypes to show greater criminogenic needs than the non-psychopathic subtype(s). Our predictions are based on previous (17, 28) and recent research (7, 25).

## Method

### Sample

The current sample consisted of 215 male violent offenders from Berlin, Germany, convicted of homicide (21.9%), sexual offenses (48.8%), or other violent offenses (predominantly assault and robbery; 29.3%) and released from prison between 1995 and 1998. The age at release varied from 19 to 59 years (*M* = 36.2, *SD* = 8.9, *n* = 213). Most of the offenders were German citizens (85.6%, *n* = 213) and not in a relationship at the time of the index offense (59.1%, *n* = 211). According to the German Federal Central Criminal Register, 16.7% of the sample had no prior convictions (*n* = 210).

### Recidivism

Official criminal records obtained from the federal crime registry were evaluated to assess recidivism. Information on recidivism was available for 212 offenders. The current study included general, violent, and sexual recidivism. Furthermore, cases of sexual and violent recidivism were coded as *severe* if they led to a conviction with a prison sentence of 2 or more years, as the German law defines these offenders as high-risk (§ 454 German Code of Criminal Procedure). Follow-up time varied from 7 to 11 years (*M* = 9.29, *SD* = 1.01).

## Measures

**Psychopathy Checklist—Revised (PCL-R).** The PCL-R (13) is a reliable and valid clinical assessment instrument for the observer rating of psychopathic personality (5). The PCL-R is scored from a semi-structured interview and a coding framework for relevant file information. The instrument includes 20 items, which can be considered to assess four correlated first-order factors: interpersonal (e.g., pathological lying, conning/manipulative); affective (e.g., shallow affect, lack of empathy); impulsive lifestyle (e.g., irresponsibility, impulsivity); and externalizing, antisocial tendencies (e.g., early behavior problems, criminal versatility). The two original factors (30) of psychopathic personality traits (Factor 1) and social deviance (Factor 2) can be regarded as second-level constructs (Interpersonal/Affective and Lifestyle/Antisocial), respectively. The items are coded on a 0-to-2 rating scale with 0 = not present, 1 = present to some extent, and 2 = fully present. Prior research supports the view of psychopathy as dimensional, not as taxonic (31, 32), indicating that individuals differ from each other in degree rather than in kind. The conventional PCL-R threshold for diagnosing psychopathy in North America is 30 points, whereas empirical research indicates that on average, samples from European countries show significantly lower PCL-R total scores [e.g., Ref. (33)]. Based on the analysis of 25 published empirical studies, Mokros et al. (34) suggested a corresponding threshold of ≥ 25 points for the diagnosis of psychopathy in German-speaking countries.

**Level of Service Inventory—Revised (LSI-R).** The LSI-R (26) is one of the most widely used assessment tools designed to identify the offenders’ risks and needs with regard to recidivism. In addition, there is a large body of literature supporting the validity of the LSI-R measure [for overview, see Ref. (27)]. The LSI-R consists of 54 items (scored as 1 = present or 0 = not present) assessing offenders across 10 domains, 1 static (Criminal History) and 9 dynamic or changeable criminogenic needs that are amenable to treatment: education/employment, financial, family/marital, accommodation, leisure/recreation, companions, alcohol/drug problems, emotional/personal, and attitudes/orientation. The LSI-R total score can range from 0 to 54, with higher scores indicating a greater recidivism risk and need for clinical intervention. According to an overview by Hare (13, p. 162) on the results from three samples, the components of the LSI-R are associated more strongly with PCL-R Factor 2 than with Factor 1.

## Coding

In order to assess the reliability of the LSI-R and PCL-R, ratings from two research assistants coded a random subsample of 30 cases each. The results showed an excellent level of inter-rater agreement, with intra-class correlations for a single measure (ICC) (35) of .96 and .92, respectively, for the LSI-R and PCL-R total scores.

Given the empirical evidence for a dimensional (and multifaceted) structure of psychopathy (36), information about meaningful subtypes may be lost by excluding subjects below a certain threshold for psychopathy. Therefore, an unselected sample of violent offenders across the full distribution of psychopathic traits was used, with total scores that varied from 0 to 33 (*M* = 13.4, *SD* = 7.0). The PCL-R ratings of each offender were based on file review only, which can result in lower PCL-R scores compared to the standard assessment approach (37). According to a meta-analysis from German-speaking countries (eight studies, total *N* = 1,419), the aggregate mean of the PCL-R total score based on file review only was 16.5 (34) and thus on par with the reference mean described for file reviews of North American male offender samples (*M* = 16.5) (13). Notably, the mean of the current sample was only slightly below the lower bound of the 95% CI (i.e., 14.2) reported for the aggregate mean of offender samples from German-speaking countries (34). The mean (*SD*) score of all offenders on the Interpersonal, Affective, Lifestyle, and Antisocial factors were 1.38 (1.55), 3.22 (2.11), 3.40 (2.45), and 4.05 (2.72), respectively.

## Data Analyses

LPA is a variant of LCA based on observed continuous rather than categorical variables. LPA is a method to identify homogeneous subgroups within a sample through maximum likelihood estimation. By virtue of information criteria and through modified likelihood ratio tests (38, 39), the optimum number of latent classes can be assessed. Nylund and colleagues (39) conducted a simulation study on the accuracy of statistical criteria for determining the number of latent classes in LPA. They found that the modified likelihood ratio test of Lo and colleagues (38) had a power of 84% for detecting the correct number of latent classes in simulated samples similar in size (*N* = 200) to the current one (*N* = 215). The corresponding statistical power of the Bayesian information criterion (BIC) (40) was estimated at 100% (39). However, the interpretation of the different LCA solutions should not rely only on statistical considerations and information criteria but also consider model parsimony, simplicity, and clarity (41). For an LCA solution to be interpretable, the mean probability of cluster membership per latent class should be .80 or above. Furthermore, particular latent classes in higher-order LCA solutions may simply represent subdivisions of uniform latent classes from solutions with fewer latent classes. In this case, the lower-order solution ought to be preferred. Finally, it is paramount that the number of latent classes obtained is meaningful.

For the analyses reported below, cases were assigned to one subtype in a mutually exclusive manner based on the maximum probabilities of latent class membership. Thus, the non-exclusive latent classes were treated like mutually exclusive clusters. Binary logistic regression analysis was used to predict the probability of recidivism risk (e.g., yes or no) for the different subtypes. Therefore, psychopathic subtype was used as a categorical predictor for the different criteria of recidivism.

To determine how the subtypes differed from one another with regard to the LSI-R subscales, Cohen’s *d* was computed as a measure of effect size (i.e., the difference between means, divided by the pooled *SD*). Values of *d* equal to or larger than 0.2, 0.5, and 0.8 can be considered as small, medium, and large effects, respectively (42). As we also were interested in how the subtypes differed from one another, a full set of pairwise comparisons on the 10 LSI-R subscales, as well as the total LSI-R score, were conducted. Data were analyzed with SPSS, version 19.0 (IBM Corporation, Somers, NY), and LPA was carried out with Mplus for Mac, version 6.12 (Muthén & Muthén, Los Angeles, CA).

## Results

### Person-Centered (LPA) Results

We used LPA to determine if homogeneous classes with relatively unique PCL-R four-factor profiles exist within a sample of male violent offenders. The LPA solutions with latent classes fit the data better than a unitary solution without latent classes (see **Table 1** for details). The likelihood ratio test (38) suggested that model fit did not improve substantially beyond a solution entailing three latent classes, whereas the BIC coefficient did not indicate any improvement in model fit at the transition from a five- to a six-latent-class solution. Therefore, based on the LPA fit statistics, previous research, as well as conceptual reasons, the intermediate solution with four latent classes was chosen for interpretation. The average latent class probabilities for allocation to the most likely class membership were substantial (.90, .84, .94, and .87), suggesting that the four latent classes represent separable variations on the PCL-R factors.

**Table 1:** Model Fit of the Latent Profile Analyses With Up to Six Latent Classes (N = 215).

	Number of Latent Classes					
	1	2	3	4	5	6
Log-Likelihood	−1,880.57	1,796.03	−1,759.05	−1,738.43	−1,720.87	−1,707.67
No. of Free Parameters	8	13	18	23	28	33
BIC ^a^	3,804.68	3,661.88	3,614.77	3,600.39	3,592.12	3,592.57
Adjusted BIC	3,779.33	3,620.68	3,557.73	3,527.51	3,503.29	3,488.00
AIC	3,777.71	3,618.06	3,554.09	3,522.87	3,497.74	3,481.34
(−2)*Log-Likelihood Difference ^b^	–	169.06	73.96	41.24	35.12	26.40
LMR LRT, *p* Value ^c, e^	–	<.001	.008	.263	.388	.034
Bootstrap LRT, *p* Value ^c, d^	–	<.001	<.001	<.001	<.001	<.001
1 – Entropy	–	.873	.828	.826	.844	.827

AIC, Akaike’s information criterion; BIC, Bayesian information criterion; LRT, likelihood ratio test. ^a^Incremental changes of BIC < 2 are considered marginal (Kass and Raftery (43), p. 777). ^b^Difference between models with (k − 1) and k classes. ^c^LMR, Likelihood ratio test according to Lo, Mendell, and Rubin (38). ^d^LRT according to Nylund et al. (39). ^e^If < .05, a model with k latent classes will fit significantly better than a model with (k − 1) latent classes.

After the assignment of cases to manifest subtypes based on the maximum allocation probability, the four subtype groups contained 38, 57, 105, and 15 individuals, respectively. We first labeled the four subtypes as *Latent Class 1* (LC1; 7% of the sample), *Latent Class 2* (LC2; 17.7% of the sample), *Latent Class 3* (LC3; 26.5% of the sample), and *Latent Class 4* (LC4; 48.8% of the sample). The mean (*SD*) PCL-R total score of each latent class was as follows: LC1, 25.8 (4.2); LC2, 18.2 (3.5); LC3, 17.4 (3.8); and LC4, 7.6 (3.4).

**Figure 1:**
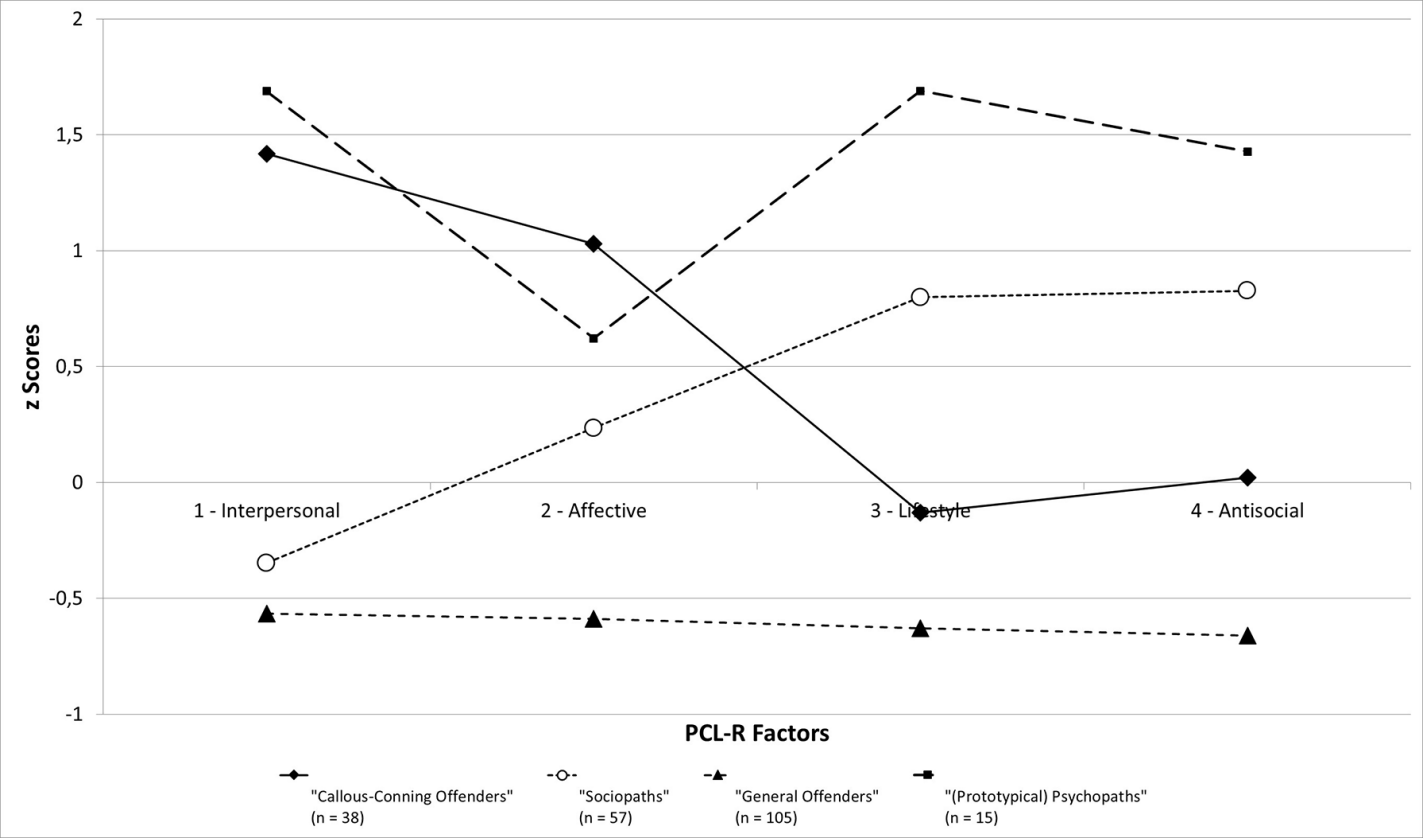
Mean z-scores of each latent class on each PCL-R factor.

For display purposes, factor scores were converted to *z*-scores, with a *z*-score of 0 representing the sample mean (see **Figure 1**). Consistent with previous research (7, 25) and current hypotheses, the four subtypes conformed to prototypic (LC1), callous-conning (LC2), sociopathic (LC3), and general offender (LC4) profiles. As **Figure 1** shows, individuals assigned to LC4 (general offenders) had low average scores on all four first-order PCL-R factors. Those allocated to LC3 (sociopathic offenders) had high mean scores on the Lifestyle and Antisocial factors of the PCL-R, yet they had average scores on the Interpersonal and Affective factors, close to the grand mean of the sample (i.e., a *z*-score of 0). In contrast, those allocated to LC2 (callous-conning offenders) displayed high mean scores on the Interpersonal and Affective factors of the PCL-R, yet they lacked high scores on the Lifestyle and Antisocial factors. Finally, the individuals allocated to LC1 (prototypical psychopaths) had the highest scores on the Interpersonal, Lifestyle, and Antisocial factors of the PCL-R.

Concerning the Affective factor, LC1 had a slightly lower mean score than did LC2, at a moderate mean difference (*d* = 0.59). This difference is somewhat at odds with other recent LPA research (5, 7, 44) and may be due to the file-only status of the PCL-R data and the likelihood that affective features were less adequately assessed in psychopathic than other offenders.

### External Validation Analyses

**Needs assessment.** Because the four subtypes were compared to each other with regard to the 11 LSI-R scales, we controlled for family-wise error by using a Bonferroni–Holm correction (45). The LSI-R total scores (**Table 2**) indicated that individuals assigned to LC4 were at a low/moderate risk to reoffend (26), which was significantly different from LC1 (a large effect, *d* = −1.92) and LC3 (a large effect, *d* = −1.84). The risk for individuals assigned to LC4 was also lower than the average risk posed by individuals assigned to LC2 (a moderate effect, *d* = −0.56). In terms of LSI-R total scores, individuals assigned to LC3 (*M* = 31.4, *SD* = 5.3) and LC1 (*M* = 32.7, *SD* = 4.7) were at the upper end of the moderate recidivism risk category (24 to 33 points).

**Table 2:** Mean (SD) Scores of the Latent Classes and Pairwise Comparisons Between Classes for Each LSI Subcomponent.

	LC4—General Offender		LC3—Sociopathic Offender		LC2—Callous-Conning Offender		LC1—(Prototypical) Psychopaths		LC4 vs. LC3	LC4 vs. LC2	LC4 vs. LC1	LC3 vs. LC2	LC3 vs. LC1	LC2 vs. LC1
LSI	*M*	*SD*	*M*	*SD*	*M*	*SD*	*M*	*SD*	*d*	*d*	*d*	*d*	*d*	*d*
Total Score	18.99	7.49	31.40	5.29	23.16	7.27	32.73	4.67	−1.84*	−0.56	−1.92*	1.35*	−0.26	−1.47*
Criminal history	3.83	2.09	6.58	1.58	5.55	2.34	7.20	1.42	−1.44*	−0.8*	−1.68*	0.54	−0.41	−0.79
Education/ employment	4.44	2.72	7.14	1.65	4.45	2.61	7.80	2.04	−1.13*	0	−1.28*	1.31*	−0.39	−1.39*
Financial	1.10	0.78	1.60	0.59	1.16	0.75	1.60	0.63	−0.68*	−0.07	−0.65	0.67	−0.01	−0.62
Family/marital	1.89	1.15	2.56	1.00	1.95	1.21	2.53	0.92	−0.62*	−0.05	−0.58	0.57	0.03	−0.53
Accommodation	0.38	0.61	0.82	0.80	0.50	0.60	0.80	0.77	−0.65*	−0.2	−0.67	0.45	0.03	−0.47
Leisure/recreation	1.53	0.65	1.91	0.34	1.50	0.69	1.73	0.59	−0.68*	0.05	−0.31	0.82	0.45	−0.36
Companions	1.46	1.21	2.56	1.15	1.42	1.24	2.47	1.25	−0.93*	0.03	−0.84	0.97*	0.08	−0.86
Alcohol/drug problem	2.30	2.48	4.68	2.54	2.47	2.02	4.73	2.22	−0.96*	−0.07	−1*	0.95*	−0.02	−1.11
Emotional/personal	1.35	1.12	2.11	0.96	1.89	1.13	2.20	1.01	−0.71*	−0.49	−0.77	0.21	−0.1	−0.28
Attitudes/orientation	0.70	0.85	1.44	1.12	2.26	1.00	1.67	0.98	−0.77*	−1.75*	−1.12	−0.78*	−0.21	0.61

d, Cohen’s d effect size measure: mean difference in pooled SD units. * p < .05 after Bonferroni–Holm correction.

There were no significant differences between individuals assigned to LC1 and LC3 with regard to LSI-R subscales. Both subtypes tended to score highest on the LSI-R subscales (except for attitudes/orientation; see **Table 2**). In addition, differences between individuals assigned to LC4 and LC2 on the LSI-R subscales were small (**Table 2**). Only in relation to the two subscales of criminal history, and attitudes/orientation offenders assigned to LC2 tended to have significantly higher scores. They also had significantly higher scores on the attitudes/orientation subscale (procriminal and antisocial attitudes, values, beliefs, and thinking) than did the sociopathic and general offenders, with a moderate effect size (**Table 2**).

**Figure 2:**
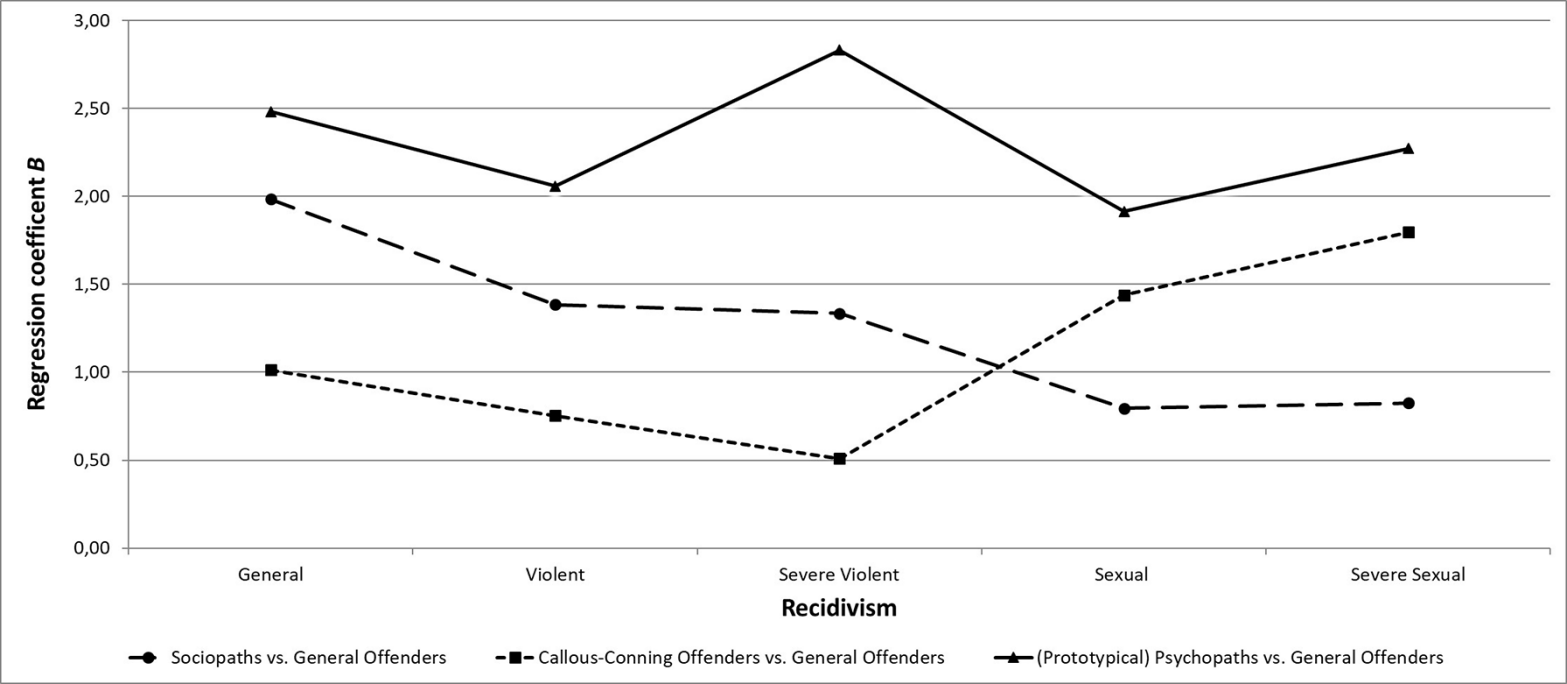
Recidivism risk profiles for psychopaths, sociopaths, and manipulative offenders compared to general offender for general, violent, severe violent, sexual, and severe sexual recidivism.

**Risk assessment.** **Figure 2** shows the recidivism profiles for the three most pathological subtypes (LC1, LC2, LC3), relative to LC4. Binary logistic regression analysis shows a clear trend for individuals assigned to LC1 to be at the highest risk to commit a new offense of any kind. In particular, for general recidivism, individuals assigned to LC1 (*B* = 2.48, *p* = .019), LC3 (*B* = 1.98, *p* < .001), and LC2 (*B* = 1.01, *p* = .019) were at significantly higher risk to commit a new offense than were individuals assigned to LC4. In terms of violent recidivism, individuals assigned to LC1 (*B* = 2.06, *p* = .001) and LC3 (*B* = 1.39, *p* = .001), but not LC2 (*B* = 0.75, *p* = .12), showed higher risk than did individuals assigned to LC4. This pattern (of higher risk compared to individuals assigned to LC4) also held up with respect to severe violent recidivism for both individuals assigned to LC3 (*B* = 1.33, *p* = .023) and LC1 (*B* = 2.83, *p* < .001) but not for individuals assigned to LC2 (*B* = 0.51, *p* = .501).

For sexual (*B* = 0.80, *p* = .146) and severe sexual (*B* = 0.83, *p* = .190) recidivism, there were no significant differences in recidivism risk between individuals assigned to LC3 and LC4. However, the risk for sexual and severe sexual recidivism was significantly higher for both individuals assigned to LC1 and LC2 than for individuals assigned to LC4. Even if non-significant, risk for sexual recidivism was higher for LCI (Factor 1 and Factor 2 traits) than for LC3 (primarily Factor 2 traits).

## Discussion

This person-oriented study used LPA with a complete sample of violent offenders and identified four latent classes. Given the high average maximum allocation probabilities for the latent classes, cases could be assigned to one of four subtypes with good accuracy. Using the terminology of previous studies (7, 25, 44), the four clusters could be designated as *prototypical psychopaths* (LC1), *callous-conning offenders* (LC2), *sociopathic or dyssocial offenders* (LC3), and *general offenders* (LC4). Here we would use the term “prototypical psychopath” descriptively and not in terms of a diagnostic category. The tentative label for latent class LC3 would be sociopathic or dyssocial rather than secondary psychopathy based on our position that the term “secondary” makes little clinical or empirical sense. For convenience, we refer to LC1 and LC3 as *psychopathic* and *sociopathic*, respectively. The emergence of a psychopathic group, a sociopathic group, a callous-conning group, and a group of offenders who are neither prototypical nor intermediate-level cases was according to expectation. In line with early clinical typologies (4, 46), individuals in the sociopathic cluster appeared dissocial without necessarily sharing the psychopath’s features of guile, lack of empathy or guilt, and emotional detachment. The callous-conning cluster (LC2) is particularly interesting, apparently sharing the manipulative skill and lack of empathy of the psychopath without displaying strong levels of impulsivity or recklessness, thereby falling short of the full expression of the psychopathy syndrome (23, 24, 47). Noteworthy is that the current four-cluster group solution is generally in line with LPAs conducted with much larger samples from North America and Europe, as described elsewhere (7, 25, 44). In this new research, based on standard interviews (plus file review), the prototypic psychopaths are the highest on all factors of PCL-R psychopathy.

Different risk assessment instruments are used in correctional and forensic-psychiatric assessments. We used the LSI-R to examine how the four subtypes might differ on risk-related criminogenic needs. The current results are in agreement with the hypothesis that psychopathic and sociopathic offenders show greater criminogenic risks and needs than other offenders (28). Here, the relative elevation of psychopathic (LC1) and sociopathic (LC3) offenders on the behavioral and social deviance features of psychopathy is consistent with previous empirical studies showing an association of Factor 2 with alcohol and drug abuse [e.g., Ref. (48)], lower educational achievement [e.g., Ref. (49)], and lower socioeconomic status (50). Also, a study using the historical, clinical, and risk management (HCR-20) (51) violence risk assessment scheme did show the highest total scores for the prototypical psychopath subgroup. Due to high levels of historical risk factors and in line with the current findings, the previous study found higher scores for the sociopathic subgroup compared to callous-conning and general offenders [compare Ref. (5)]. The fact that callous-conning offenders score significantly higher on the attitudes/orientation subscale (procriminal and antisocial attitudes, values, beliefs, and thinking) could indicate that they believe that the norms of society should not apply to them.

Recidivism risk varied as a function of offender subtype. Nearly all of the psychopathic offenders and the majority of sociopathic offenders reoffended. Overall, psychopathic offenders showed the highest risk for recidivism regardless of the criterion (i.e., general, violent, sexual). Thus, the Factor 1 components appear to add additional risk for recidivism, given that the prototypic offenders exceeded the sociopathic offenders on Factor 1 but were similar on Factor 2. At the same time, sociopathic and psychopathic offenders (each with a relative elevation on Factor 2) showed similar (higher) recidivism risk in terms of general and violent reoffending than did general offenders. The current findings are in agreement with Poythress and colleagues (20), who also did not find a significant difference in general and violent recidivism between primary and secondary psychopaths.

The relationship between PCL-R subtype classes and sexual recidivism involved results worthy of highlighting. For sexual and severe sexual recidivism, there were no significant differences in recidivism risk between sociopathic and general offenders. However, the risk for sexual and severe sexual recidivism was significantly higher for both psychopathic and callous-conning offenders than for general offenders. This is in line with findings by Krstic et al. (25) showing the callous-conning subtype to have the highest paraphilic factor scores. In agreement with Krstic et al. (25), we would argue that “high sexualization might be more related to the affective and interpersonal characteristic of psychopathy” (p. 18). While Olver and colleagues (19) found that secondary variants (e.g., LC3) had higher rates of sexual violence than did the primary subtype (e.g., LC1), one could argue, based on the results of Mokros et al. (6), that the secondary subtypes in the Olver et al. study may be better conceptualized as aggressive primary psychopathy subtypes.

The current findings may have implications for the issue of treatment amenability. Research by Durbeej and colleagues (52) and by Swogger and colleagues (53) indicates that traditional treatments are ineffective with offenders who score high on PCL-R Factor 1, especially its Affective component. Similarly, offenders with high PCL-R scores tend to drop out of treatment early (54, 55), while the Affective component is predictive of violence. This suggests that the psychopathic (LC1) and callous-conning (LC2) latent classes identified in this and other studies may include the offenders who pose the greatest challenges to treatment providers. The person-oriented research described here should prove to be a valuable addition to the more traditional variable-oriented research on psychopathy (7). For example, high PCL-R scores in combination with sexual deviance are predictive of sexual offending (56). It would be interesting to determine how sexual deviance interacts with the latent profiles described here, with sexual recidivism, as well as with treatment outcome and violence.

## Future Research

The use of file-only ratings for the PCL-R assessment likely truncated the range of scores. Similarly, the use of official records as the sole outcome measure of offense recidivism presumably underestimated the actual rate of reoffending. Accordingly, future research should replicate the current results using the standard procedure (i.e., semi-structured interview, file, and collateral information).

The fact that the psychopathic cluster (arguably the most interesting one) consisted of only 15 individuals (7% of the sample) may raise concerns about the stability of the findings and the likelihood of replication in a new sample. However, a similar profile has been identified in very large samples from both the US and Sweden (5, 7, 44).

Even though the LPA model with four latent classes was replicated in different male offender samples (e.g., violent offenders, sex offenders), psychiatric samples, and samples from different countries (North America, United Kingdom, Netherlands, Germany, Sweden), and validated using different criterion variables (e.g., offense behavior, recidivism risk, criminogenic needs), future research should extend the cross-cultural and validation research using the full PCL-R distribution.

## Ethics Statement

This study was carried out in accordance with the recommendations of the ethics committee of the Charité-Universitätsmedizin Berlin Campus Benjamin Franklin. Data collection and analyses were file-based in retrospect. The protocol was approved by Charité-Universitätsmedizin Berlin.

## Author Contributions

All authors have contributed to the manuscript and agreed to authorship in the indicated order.

## Conflict of Interest Statement

RDH receives royalties from the sale of the PCL-R and its derivatives, and income from PCL-R training workshops. AM receives royalties from the sale of the German PCL-R version, and income from PCL-R training workshops.

The remaining authors declare that the research was conducted in the absence of any commercial or financial relationships that could be construed as a potential conflict of interest.
